# The Simon Effect Based on Allocentric and Egocentric Reference Frame: Common and Specific Neural Correlates

**DOI:** 10.1038/s41598-019-49990-5

**Published:** 2019-09-24

**Authors:** Hui Li, Nan Liu, You Li, Ralph Weidner, Gereon R. Fink, Qi Chen

**Affiliations:** 1 Center for Studies of Psychological Application and School of Psychology, South China Normal University, Guangzhou, 510631 China; 2 Cognitive Neuroscience, Institute of Neuroscience and Medicine (INM-3), Research Center Jülich, 52425 Jülich, Germany; 3 Department of Neurology, University Hospital Cologne, 50937 Cologne, Germany; 4 Guangdong Key Laboratory of Mental Health and Cognitive Science, South China Normal University, Guangzhou, 510631 P.R. China

**Keywords:** Cognitive control, Human behaviour

## Abstract

An object’s location can be represented either relative to an observer’s body effectors (egocentric reference frame) or relative to another external object (allocentric reference frame). In non-spatial tasks, an object’s task-irrelevant egocentric position conflicts with the side of a task-relevant manual response, which defines the classical Simon effect. Growing evidence suggests that the Simon effect occurs not only based on conflicting positions within the egocentric but also within the allocentric reference frame. Although neural mechanisms underlying the egocentric Simon effect have been extensively researched, neural mechanisms underlying the allocentric Simon effect and their potential interaction with those underlying its egocentric variant remain to be explored. In this fMRI study, spatial congruency between the task-irrelevant egocentric and allocentric target positions and the task-relevant response hand was orthogonally manipulated. Behaviorally, a significant Simon effect was observed for both reference frames. Neurally, three sub-regions in the frontoparietal network were involved in different aspects of the Simon effect, depending on the source of the task-irrelevant object locations. The right precentral gyrus, extending to the right SMA, was generally activated by Simon conflicts, irrespective of the spatial reference frame involved, and showed no additive activity to Simon conflicts. In contrast, the right postcentral gyrus was specifically involved in Simon conflicts induced by task-irrelevant allocentric, rather than egocentric, representations. Furthermore, a right lateral frontoparietal network showed increased neural activity whenever the egocentric and allocentric target locations were incongruent, indicating its functional role as a mismatch detector that monitors the discrepancy concerning allocentric and egocentric object locations.

## Introduction

The human brain codes objects that are present in a visual scene simultaneously and in different spatial coordinate systems. For example, the spatial location of an object can be represented in an egocentric frame, i.e., its position is encoded with reference to the body effectors of the observer. Alternatively, an object’s location can be represented in an allocentric frame, i.e., relative to an object or a background, independent of its egocentric position relative to the observer, such as the right or left side of an object in the outside world. These different types of representations constitute the basis for specific subsequent cognitive processes. In particular, egocentric representations are proposed to underlie goal-directed actions^1–4^, while allocentric representations are proposed to support conscious perception of objects or spatial memory functions^1,2,5,6^.

Accumulating previous evidence indicated that an object’s location is automatically coded and cannot be ignored irrespective of whether or not it is irrelevant to a current behavioral task^7^. For example, in the classical Simon effect, subjects are requested to perform a discrimination task about a non-spatial object feature, such as color, luminance, shape, or sound, of a target, and indicate the respective feature by a manual response. In particular, a single feature is consistently mapped to either a left or a right manual response^8–10^. Although the egocentric position of the object (left/right) is irrelevant to the non-spatial discrimination task, it influences task performance depending on whether or not the target position is spatially consistent with the side of the response. Responses are slower when the irrelevant egocentric position of the target is incompatible with the side of the pre-defined manual response and quicker when both are compatible. This Simon effect has been observed across a variety of experimental manipulations, including the type of stimulus (e.g., auditory and visual stimulus^11^), spatial arrangement (e.g., vertical and horizontal presentation^12^), and reaction mode (e.g., cross hand response^13^; vocal response^14^). Neural mechanisms underlying the egocentric Simon effect have been found in the frontal-striatal cortex, anterior cingulate cortex (ACC), the pre-supplementary and the supplementary motor areas (pre-SMA/SMA), and superior as well as inferior parietal gyrus (IPG)^15–20^. Interestingly, growing evidence suggests that the Simon effect is not only based on egocentric but also on allocentric coordinates^10,21^. For instance, responses are slower when the allocentric location of the target - although completely task-irrelevant - and the side of response hand are incongruent compared to when both are congruent. Behaviorally, the allocentric Simon effect has been demonstrated to be independent of the egocentric Simon congruency^10^.

**Figure 1:**
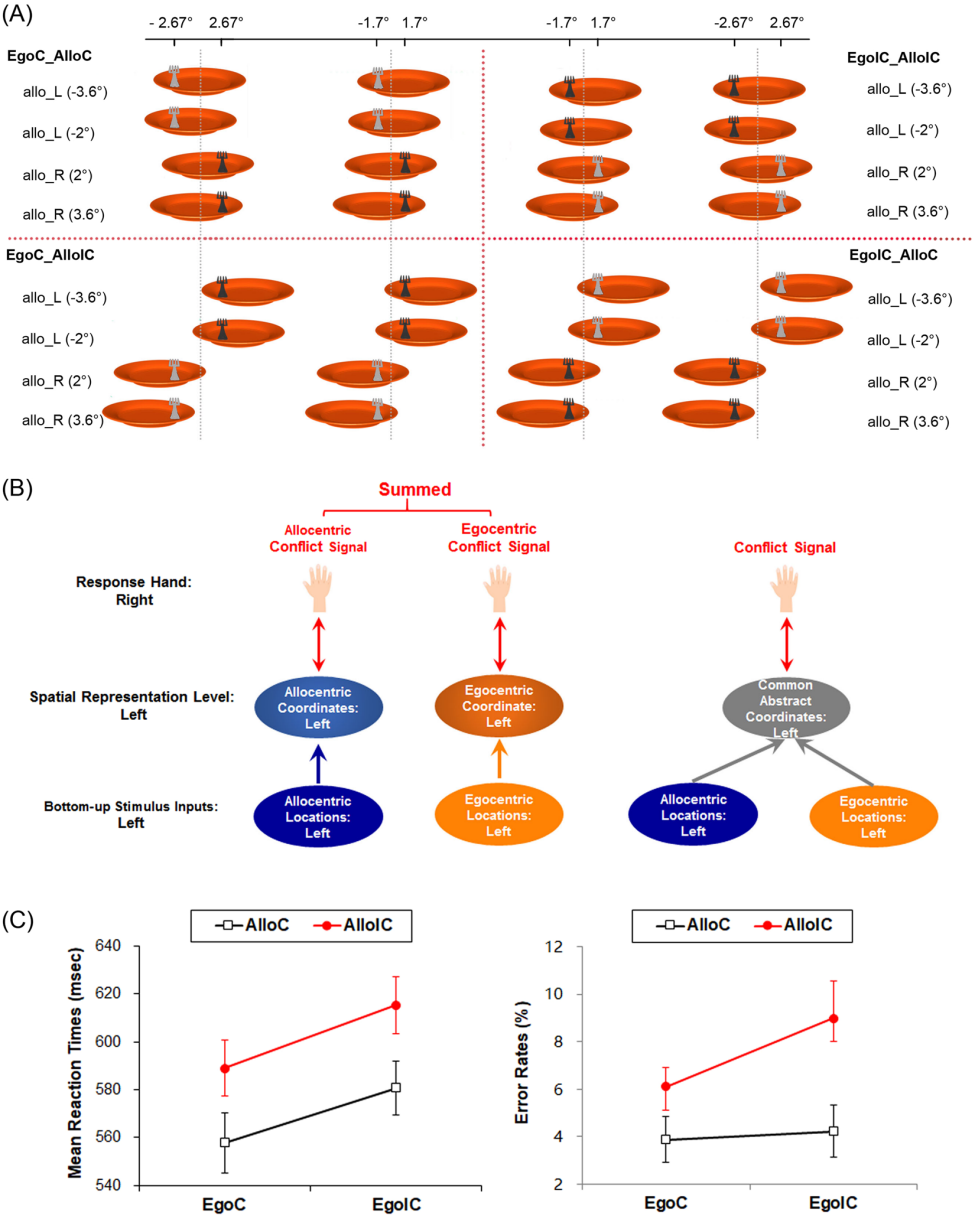
(**A**) Experimental stimuli. The target stimulus is the fork, with two levels of luminance. The gray dotted line represents the invisible egocentric mid-sagittal plane. The four egocentric locations (−2.67°, −1.7°, 1.7°, 2.67°) of the fork are marked on the top of the figure, and the four allocentric locations (−3.6°, −2°, 2°, 3.6°) of the fork (with reference to the mid-sagittal plane of the plate) are marked on the left and right side of the figure. For the examples of the four experimental conditions defined here, participants were asked to respond to the light grey via their left hand, and to the dark grey via their right hand. The bottom-up stimuli are fully counter-balanced for the three critical contrasts in the present experiment, i.e., the main effect of egocentric congruency “EgoC (AlloC + AlloIC) vs. EgoIC (AlloC + AlloIC)”, the main effect of allocentric congruency “AlloC (EgoC + EgoIC) vs. AlloIC (EgoC + EgoIC)”, and the interaction contrast “EgoC (AlloC > AlloIC) vs. EgoIC (AlloC > AlloIC)”. (**B**) Two hypothetical models of the neural interaction between the egocentric and allocentric Simon effect. (**C**) Behavioral results. Mean RTs (ms) and mean error rates (%) with standard errors in the four experimental conditions.

It remains to be investigated, whether the neural mechanisms underlying the allocentric Simon effect are different from those involved in generating its egocentric variant or whether both share common resources. In the current fMRI study, to answer this question, we orthogonally combined the Simon effects based on the egocentric and allocentric reference frames with a non-spatial luminance discrimination task (Fig. 1A). The allocentric position of the behavioral target could be either consistent or inconsistent with the side of the response hand, irrespective of the spatial congruency between the target’s egocentric position and the side of response hand. Therefore, the current factorial design resulted in four experimental conditions: (1) an egocentric congruent and allocentric congruent condition (EgoC_AlloC); (2) an egocentric congruent and allocentric incongruent condition (EgoC_AlloIC); (3) an egocentric incongruent and allocentric congruent condition (EgoIC_ AlloC); and (4) an egocentric incongruent and allocentric incongruent condition (EgoIC_AlloIC) (Fig. 1A). This factorial design allows determining the main effects of spatial congruency within each reference frame, and hence localizing the neural mechanisms of the Simon effect induced within different reference frames. Furthermore, the factorial design allows revealing the neural interactions between the two types of Simon effects.

Brain regions that play a role in resolving conflicts induced by the Simon task are expected to show increased activation related to incongruent stimulus-response mappings, independent of whether these were induced by allocentric or egocentric coding of object locations. Furthermore, we were interested in two possible patterns of interaction between the neural processes underlying the egocentric and allocentric Simon effect. First, if the two types of Simon effect occur based on discrete spatial coordinate systems specific to the allocentric and egocentric representations, the two concrete conflict signals originating from the two specific spatial coordinate systems should be additive (Fig. 1B, left)^22–24^. Accordingly, neural activity in the double-source incongruent (EgoIC_AlloIC) condition should be additive, i.e., equal to the summed activity of the two single-source incongruent (EgoIC_AlloC and EgoC_AlloIC) conditions. Alternatively, the two types of Simon conflict could occur based on a common higher-level or abstract spatial coordinate system, in which the allocentric and egocentric positions might be coded in an integrated fashion within the same abstract coordinate system (Fig. 1B, right). Since both share an abstract coordinate map, the conflict signal in the double-source incongruent (EgoIC_AlloIC) condition would be generated based on the same abstract incongruent coordinate as that in the two single-source incongruent conditions. Therefore, incongruent neural activity should be comparable between the three incongruent conditions.

More critically, in the congruent condition (EgoC_AlloC) and the double-source incongruent condition (EgoIC_AlloIC), the allocentric and egocentric positions of the target were on the same side, i.e., matched (Fig. 1A), while in the two single-source incongruent conditions (i.e., EgoC_AlloIC and EgoIC_AlloC), the allocentric and egocentric positions of the target were on the opposite side, i.e., mismatched (Fig. 1A). We accordingly hypothesized that, in the present study, there should be specific brain mechanisms involved in monitoring the mismatch of spatial representations about different spatial reference frames, rather than resolving the Simon conflict per se. In particular, the mismatch monitor should be significantly activated by the two single-source incongruent conditions (EgoC_AlloIC and EgoIC_AlloC), in which the egocentric and allocentric locations mismatched, compared with the congruent (EgoC_ AlloC) and the double-source incongruent (EgoIC_ AlloIC) condition, in which the two sources of spatial representations matched.

## Materials and Methods

### Participants

Forty right-handed healthy volunteers (22 females and 18 males, 18–25 years old) with no history of neurological, psychiatric, or color vision impairments took part in the current fMRI study. They all had a normal or corrected-to-normal vision. The study was designed and conducted following the guidelines of the Helsinki Declaration. All participants had given informed consent for the study before the experiment and got paid for their participation after the experiment. The Ethics Committee of the School of Psychology, South China Normal University, had approved the experimental procedures.

### Stimuli and experimental setup

The target stimulus was a fork (2.5° of visual angle in width), placed on the top of an orange plate (15° of visual angle in diameter), with a gray background (Fig. 1A). The color of the fork was either light (RGB value: 64, 64, 64) or dark (RGB value: 192, 192, 192) gray in each trial. As shown in Fig. 1A, the fork had 4 allocentric positions with reference to the median sagittal axis of the plate (i.e., −3.6°, −2°, 2°, and 3.6°) and 4 egocentric positions with reference to the median sagittal axis of the participants’ body (i.e., −2.67°, −1.7°, 1.7°, and 2.67°) (Fig. 1A). The egocentric locations of the fork were orthogonally varied to the allocentric locations. The visual angles of the egocentric and allocentric positions of the target were chosen using a psychophysical test before the formal experiment, to balance the difficulty between the two judgment tasks.

### Experimental design and tasks

It has been suggested that participants tended to use the egocentric reference frame even when they were not explicitly instructed which specific frame to adopt^25^. Previous evidence from our lab further suggested that the allocentric Simon effect occurred only when the current task demands explicitly induced allocentric representations while the egocentric Simon effect existed irrespective of whether the egocentric representations were explicitly coded or not^10^. Therefore, to make sure that both the egocentric and allocentric Simon effects occur in the present experiment, we adopted two additional spatial judgment tasks (i.e., egocentric and allocentric judgment tasks), and alternated them with the main luminance discrimination task, to explicitly elicit the coding of the egocentric and allocentric representations. Specifically speaking, subjects were asked to perform three tasks on the same stimuli. First, participants were required to judge whether the position of the target was left or right respect to the median sagittal axis of their own body (egocentric judgment). Second, subjects were asked to judge whether the position of the target was left or right respect to the median sagittal axis of the plate (allocentric judgment task). Third, participants needed to discriminate whether the luminance type of the target was light or dark (luminance discrimination task). Currently, we focused our analysis on the non-spatial luminance discrimination tasks. Two response pads on each side of the participants’ body were used. Participants were required to press a button on the left pad using their left thumb corresponding to the left side judgment or the dark gray judgment, and vice versa. The mapping between the luminance type and the response side was counterbalanced across all participants. The spatial judgment tasks and the non-spatial judgment tasks were alternated on a block-by-block basis. A luminance discrimination block followed every spatial judgment block. An instruction was displayed before each block for 3 seconds, informing the participants of the task in the upcoming block.

Moreover, in the luminance task, we manipulated the congruency between the task-irrelevant egocentric and allocentric positions of the target and the side of the response. The two types of the task-irrelevant spatial position of the fork could be either congruent or incongruent with the response side. Therefore, the experimental design about the luminance discrimination task was a 2 (egocentric congruency: incongruent vs. congruent) × 2 (allocentric congruency: incongruent vs. congruent) within-subject design. The luminance discrimination task had 12 blocks. Each block contained 16 experimental trials and 5 to 6 null trials (only a blank screen was displayed). Within each block, the order of trials was randomized for each participant to avoid potential expectations. The target was presented for 250 ms in each trial. We chose such a short duration for minimizing unnecessary eye movements^26^. During the whole experiment, participants were asked to keep their eyes looking straightforward without moving them. The duration of each trial was jittered from 2000 ms to 3000 ms with a step of 250 ms.

### Data acquisition

All MR images were acquired by a 3 T Siemens Trio system with a standard 32-channel head coil. The following scanning parameters were used for T2*-weighted echo-planar images (EPI): TR = 2.2 s, TE = 30 ms, FA = 90°, FOV = 200 mm, matrix = 64 × 64, voxel size = 3.4 × 3.4 × 3 mm^3^. We acquired 36 transversal slices covering the whole brain with a 0.75 mm gap. The functional scanning session resulted in 636 EPI volumes, which lasted for 23.32 minutes. After the functional scanning, high-resolution images (voxel size = 1 × 1 × 1 mm^3^) of the whole brain were acquired for anatomic co-registration, using a standard T1-weighted 3D sequence (MPRAGE).

### Statistical analysis of behavioral data

For each experimental condition, trials with incorrect responses or with reaction times (RTs) outside three times standard deviation (SD) were excluded from further analysis. Error rates were the percentage of excluded trials under each experimental condition. Mean RTs and error rates were then entered into a 2 (egocentric congruency: incongruent vs. congruent) × 2 (allocentric congruency: incongruent vs. congruent) repeated-measures analyses of variance (ANOVA), respectively.

### Statistical analyses of imaging data

All image data were processed and analyzed using the general linear model (GLM) in SPM12 (Wellcome Department of Imaging Neuroscience, London, http://www.fil.ion.ucl.ac.uk). First, the first 5 volumes were deleted, and the remaining images were realigned to the new first volume to correct for inter-scan head movement. Then, images were normalized to the standard MNI space and resampled to 2 × 2 × 2 mm^3^ voxel size. Finally, the normalized images were smoothed (8 mm FWHM) to increase the signal/noise ratio in the images. Data were high-pass-filtered at 1/200 Hz.

At the first level, the GLM was used to construct a multiple regression design matrix. The four critical experimental conditions in the luminance discrimination tasks were modeled: “EgoC_AlloC”, “EgoC_AlloIC”, “EgoIC_AlloC”, and “EgoIC_AlloIC”. Another two regressors were additionally modeled in the GLM, accounting for variances induced by all the trials in the egocentric and allocentric spatial judgment tasks, respectively, which were independent of the four critical regressors in the non-spatial luminance discrimination tasks. All the event types were time-locked to the onset of the target of each trial by a standard HRF and its first-order time derivative (TD) with an event duration of 0 s. All the instructions, the error trials, and the six head movement parameters derived from the realignment procedure were included as covariates of no interest. Temporal autocorrelation was modeled using an AR (1) process. Parameter estimates were calculated for each voxel using weighted least-squares to provide maximum likelihood estimators based on the temporal autocorrelation of the data. For each participant, simple main effects for each of the 4 experimental conditions were computed by applying appropriate ‘1 0’ baseline contrasts (experimental conditions vs. null trials). At the second group level, the 4 first-level individual contrast images were entered into a 2 × 2 within-subject ANOVA by employing a random-effects model. In the modeling of variance components, we allowed for violations of sphericity by modeling non-independence across parameter estimates from the same participant and allowed for unequal variances both between conditions and between participants using the standard implementation in SPM12.

**Figure 2:**
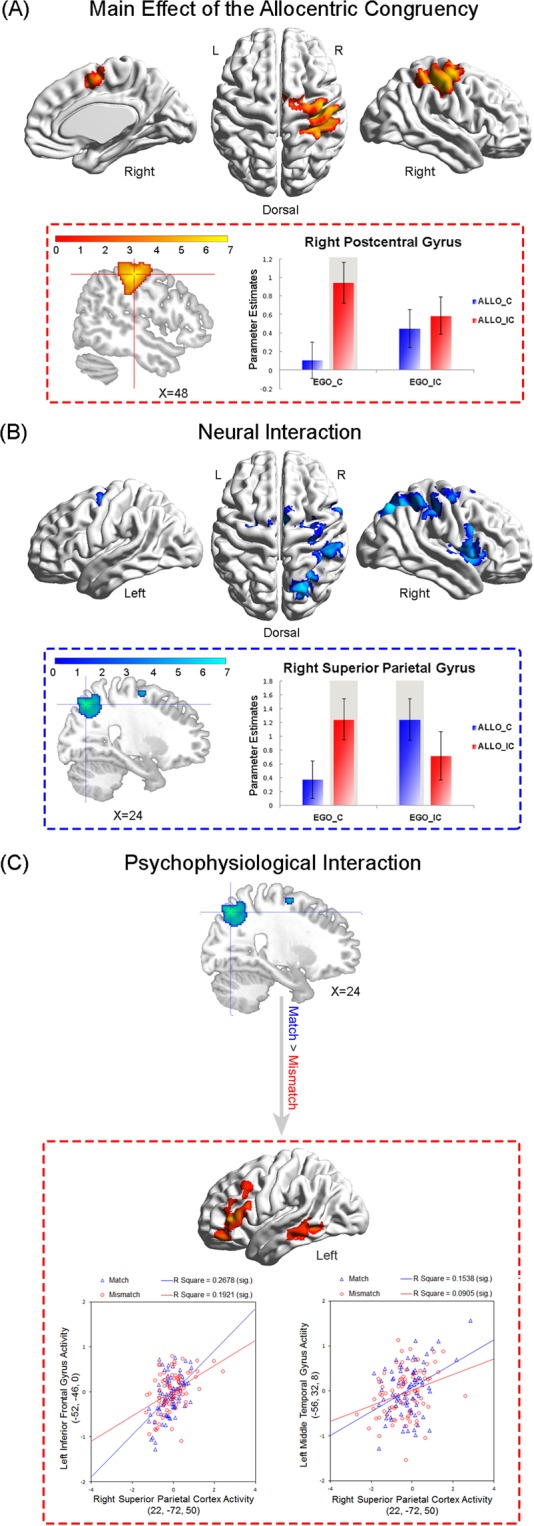
(**A**) Main effect of the allocentric congruency. Brain areas activated by allocentric incongruent conditions relative to the allocentric congruent conditions irrespective of the egocentric congruency, i.e., AlloIC (EgoC + EgoIC) > AlloC (EgoC + EgoIC). Mean parameter estimates extracted from the activated cluster are shown as a function of the four experimental conditions. (**B**) Neural interactions between the egocentric and the allocentric congruency, i.e. the neural interaction contrast “EgoC (AlloIC > AlloC) > EgoIC (AlloIC > AlloC)”. Mean parameter estimates extracted from the activated cluster are shown as a function of the four experimental conditions. Error bars indicate SEs. (**C**) PPI analysis based on neural activity in the right SPG (MNI: 24, −70, 48) with the contrast “Match vs. Mismatch” as the psychological factor. The right SPG (upper panel) showed enhanced functional connectivity with the left IFC and the left MT (lower panel) in the “Match” conditions than in the “Mismatch” conditions. For a representative participant, mean corrected neural activity in the left IFC (lower left panel) and the left MT (lower right panel) was plotted as a function of the mean corrected neural activity in the right SPG in the “Match” and “Mismatch” conditions, respectively.

First, we performed the conventional ANOVA analysis on the present factorial design, i.e., the main effect of allocentric congruency [“AlloIC (EgoC + AlloIC) > AlloC (EgoC + AlloIC)”, and vice versa], the main effect of egocentric congruency [“EgoIC (AlloC + AlloIC) > EgoC (AlloC + AlloIC)”, and vice versa], and the interaction between allocentric and egocentric congruency [“EgoC (AlloIC > AlloC) > EgoIC (AlloIC > AlloC)”, and vice versa]. The main effect contrasts are supposed to localize the specific neural activations involved in the congruency effect concerning one spatial reference frame, irrespective of the spatial congruency in the other reference frame. However, the present results showed that the allocentric and the egocentric Simon effect in the frontoparietal network could not be cleanly teased apart by the conventional analysis of the neural main effects (Fig. 2A). Rather, the significant main effect of the allocentric congruency seemed to be driven by an interaction effect (Fig. 2A): neural activity significantly increased in the ALLO_IC, compared to the ALLO_C condition, only in the EGO_C condition, but not in the EGO_IC condition. Therefore, the conventional analysis of main effects would not allow us to precisely localize the specific neural mechanisms underlying the allocentric vs. egocentric congruency effect. To further isolate the specific neural correlates underlying the different types of Simon conflicts, we directly compared the three incongruent conditions and localized the cortical regions that showed significantly higher neural activity in one incongruent condition, compared to the other two incongruent conditions. Specifically, “(EgoC_AlloIC) * 2 > [(EgoIC_AlloC) + (EgoIC_AlloIC)]” was calculated to localize the single-source allocentric Simon conflict, “(EgoIC_AlloC) * 2 > [(EgoC_AlloIC) + (EgoIC_AlloIC)]” was calculated to localize the single-source egocentric Simon conflict, and “(EgoIC_AlloIC) * 2 > [(EgoC_AlloIC) + (EgoIC_AlloC)]” was calculated to localize the double-source Simon conflict induced by both frames.

Moreover, to localize the common neural correlates between the three types of Simon conflicts, we first calculated the specific pattern of neural activity elicited by the three incongruent conditions, as compared to the congruent condition, i.e., “EgoC_AlloIC > EgoC_AlloC”, “EgoIC_AlloC > EgoC_AlloC”, and “EgoIC_AlloIC > EgoC_AlloC”. Subsequently, a statistical conjunction was performed between the above three contrasts, i.e., “(EgoC_AlloIC > EgoC_AlloC) ∩ (EgoIC_AlloC > EgoC_AlloC) ∩ (EgoIC_AlloIC > EgoC_AlloC)”. The conjunction null hypothesis, instead of the global null hypothesis, was tested for the conjunction analyses^18,27^. For all statistical analyses, areas of activation were identified as significant only if they passed a threshold of p < 0.001, family-wise error (FWE) correction for multiple comparisons at the cluster level, with an underlying voxel level of p < 0.001 uncorrected^28^.

**Figure 3:**
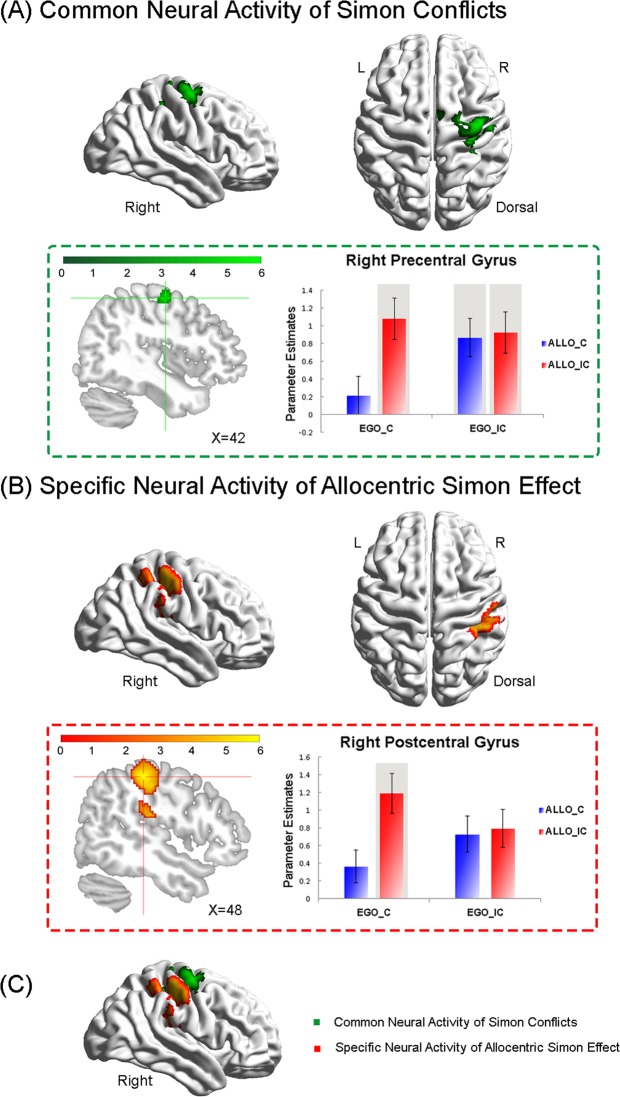
(**A**) Common neural correlates underlying the single-source and the double-source incongruent conditions. The right precentral gyrus was activated by the conjunction analysis between the single-source allocentric conflict, i.e., EgoC (AlloIC > AlloC), the single-source egocentric conflict, i.e., AlloC (EgoIC > EgoC), and the double-source conflict, i.e., EgoIC_AlloIC > EgoC_AlloC. Mean parameter estimates extracted from the activated cluster are shown as a function of the four experimental conditions. (**B**) Specific neural correlates underlying the allocentric Simon conflict. Parameter estimates were extracted from the activated clusters in the right PosCG, and are shown as a function of the four experimental conditions. Error bars indicate SEs. (**C**) Overlay of the activated brain regions in (**A**) (green) and (**B**) (orange).

Three regions of interest (ROI) were defined based on the results of group analysis: (1) the right precentral gyrus (PreCG; MNI: 42, −10, 56; BA6), which was generally activated by the Simon conflicts “(EgoC_AlloIC > EgoC_AlloC) ∩ (EgoIC_AlloC > EgoC_AlloC) ∩ (EgoIC_AlloIC > EgoC_AlloC)”, no matter which spatial reference frame was involved (Fig. 3A and Table 2A); (2) the right postcentral gyrus (PosCG; MNI: 48, −28, 50; BA2), which was specifically activated by the allocentric Simon conflict “(EgoC_AlloIC) * 2 > [(EgoIC_AlloC) + EgoIC_AlloIC)]” (Fig. 3B and Table 2B); and (3) the posterior portion of the right superior parietal gyrus (posterior SPG; MNI: 24, −70, 48; BA7), which was involved in the neural interaction contrast “EgoC (AlloIC > AlloC) > EgoIC (AlloIC > AlloC)” (Fig. 2B and Table 1B). In the three ROIs, mean parameter estimates of the critical condition were further extracted using MarsBar 0.44 (http://sourceforge.net/projects/marsbar). No further ANOVA was performed on the extracted parameter estimates to avoid the problem of double dipping^29,30^. The figures of the extracted parameter estimates in Figs 2 and 3 were drawn only for demonstration purposes.

**Table 1:** (A) Main effects of allocentric congruency. (B) The neural interaction between egocentric and allocentric congruency. (C) Brain regions that showed higher functional connectivity with the right superior parietal cortex in the “Match” than “Mismatch” condition. The coordinates (x, y, z) correspond to MNI coordinates. Displayed are the coordinates of the maximally activated voxel within a significant cluster as well as the coordinates of relevant local maxima within the cluster (in Italics).

Anatomical region	Side	Cluster peak (mm)	*t-Score*	*kE (voxels)*
***(A) AlloIC (EgoC*** + ***EgoIC*)** > ***AlloC (EgoC*** + ***EgoIC)***				
Postcentral gyrus	R	48, −22, 52	6.80	2776
*Precentral gyrus*	*R*	*42*, *−10*, *58*	*6*.*42*	
***(B) EgoC (AlloIC*** > ***AlloC)*** > ***EgoIC (AlloIC*** > ***AlloC)***				
Superior parietal *gyrus*	R	24, −70, 48	5.46	1911
*Inferior parietal gyrus*	*R*	*48*, *−32*, *50*	*4*.*93*	
*Postcentral gyrus*	*R*	*44*, *−28*, *42*	*4*.*25*	
Supplementary motor area	R	10, 2, 60	5.32	566
*Superior frontal gyrus*	*L*	*−16*, *6*, *52*	*4*.*51*	
*Supplementary motor area*	*L*	*−10*, *4*, *52*	*4*.*30*	
Cerebellum	L	−6, −66, −20	5.20	383
Rolandic operculum	R	52, −6, 8	4.74	875
*Precentral gyrus*	*R*	*58*, *8*, *26*	*4*.*63*	
Superior frontal gyrus	R	26, −6, 62	4.37	337
*Precentral gyrus*	*R*	*34*, *−16*, *60*	*4*.*33*	
***(C) PPI results***				
Inferior frontal gyrus	L	−52, 36, 8	6.64	612
Middle temporal gyrus	L	−52, −46, 0	5.10	332

**Table 2:** (A) Common neural activity underlying the Simon conflicts, irrespective of the spatial reference frame involved. (B) Specific conflict-related activity of the allocentric Simon effect. The coordinates (x, y, z) correspond to MNI coordinates. Displayed are the coordinates of the maximally activated voxel within a significant cluster as well as the coordinates of relevant local maxima within the cluster (in Italics).

Anatomical region	Side	Cluster peak (mm)	*t*-Score	*k*_E_ (voxels)
***(A) Conjunction: (EgoC_AlloIC*** > ***EgoC_AlloC)*** ∩ ***(EgoIC_AlloC*** > ***EgoC_AlloC)*** ∩ ***(EgoIC_AlloIC*** > ***EgoC_AlloC)***				
Precentral gyrus	R	42, −10, 56	5.19	1010
*Precentral gyrus*	*R*	*30*, *−24*, *52*	*4*.*54*	
*Postcentral gyrus*	*R*	*30*, *−36*, *46*	*4*.*17*	
*Supplementary motor area*	*R*	*8*, *0*, *54*	*4*.*12*	
(B) (EgoC_AlloIC) * 2 > ***(EgoIC_AlloC*** + ***EgoIC_AlloIC)***				
Postcentral gyrus	R	48, −28, 50	5.97	937
*Postcentral gyrus*	*R*	*42*, *−30*, *44*	*5*.*21*	
*Inferior Parietal gyrus*	*R*	*36*, *−40*, *50*	*4*.*27*	
*Supramarginal gyrus*	*R*	*36*, *−36*, *44*	*4*.*14*	

### Psychophysiological interaction (PPI) analysis

According to the results of neural interaction contrast “EgoC (AlloIC > AlloC) > EgoIC (AlloIC > AlloC)”, the activation of the right SPG significantly increased when the egocentric and allocentric position mismatched (i.e. one left and one right) in the EgoC_AlloIC and EgoIC_AlloC conditions than when matched (i.e. both left or both right) in the EgoC_AlloC and EgoIC_AlloIC conditions (Fig. 2B and Table 1B). To further investigate how the right SPG was involved in monitoring the “match vs. mismatch” in spatial locations relative to the two spatial reference frames, the right posterior SPG was used as a source region (MNI: 24, −70, 48; Fig. 2B and Table 1B) to estimate the context-specific functional modulation of neural activity across the brain, using PPI analysis. The neural activity in the right SPG was used as the physiological factor, and the contrast “Mismatch (EgoC_AlloIC + EgoIC_AlloC) vs. Match (EgoC_AlloC + EgoIC_AlloIC)”, which is identical to the neural interaction contrast, was used as the psychological factor.

First, the neural contrast “Mismatch > Match” was calculated at the individual level. Then, individual peak voxel was determined as the maximally activated voxel within a sphere of 16 mm radius (i.e., twice the smoothing kernel) around the peak voxel within the right posterior SPG (MNI: 24, −70, 48) for each participant. All individual peak voxels were located in the right SPG (x = 24 ± 8, y = −70 ± 6, z = 48 ± 6). Next, time series were extracted from a sphere of 4 mm radius (twice the voxel size) around the individual peak voxels within the right SPG. PPI analysis at the individual level employed three regressors: (1) The physiological variable of interest (i.e. the time series extracted from the right SPG); (2) The psychological variable of interest (i.e. “Mismatch vs. Match”), (3) The cross product of the previous two (i.e. the PPI term). An SPM was calculated to reveal brain areas in which the neural activation was predicted by the PPI term, with the physiological and the psychological regressors being treated as confound variables, i.e., by putting 1 on the PPI regressor and 0 on the physiological and the psychological regressors, respectively. At the group level, the random-effects analysis was adopted: the individual SPMs corresponding to the PPI term of each participant were subsequently fed into a one-sample *t-*test (*p* < 0.001, FWE correction at the cluster level, with an underlying voxel level of *p* < 0.001, uncorrected).

## Results

### Behavioral results

For RTs, the main effect of egocentric congruency was significant, *F*_(1,39)_ = 32.95, *p* < 0.001, indicating that participants responded significantly slower in the EgoIC condition (598 ± 11 ms) than in the EgoC (573 ± 12 ms) condition, i.e., a significant Simon effect based on egocentric locations. The main effect of allocentric congruency was significant as well, *F*_(1,39)_ = 62.72, p < 0.001, indicating that participants responded significantly slower in the AlloIC condition (602 ± 11 ms) than in the AlloC condition (569 ± 12 ms), i.e., a significant Simon effect based on allocentric locations. The two-way interaction was not significant, *F*_(1,39)_ < 1 (Fig. 1C, left).

For error rates, the pattern of results was similar to that of RTs. The main effect of egocentric congruency was significant, *F*_(1,39)_ = 4.13, *p* < 0.05, with participants making more errors in the EgoIC condition (6.6% ± 1%) than in the EgoC condition (5.0% ± 0.9%). The main effect of allocentric congruency was also significant, *F*_(1,39)_ = 12.51, *p* < 0.01, with participants making more errors in the AlloIC (7.6% ± 1.2%) condition than in the AlloC (4.1% ± 0.7%) condition. The interaction between allocentric and egocentric congruency was not significant, *F*_(1,39)_ = 2.65, *p* = 0.11 (Fig. 1C, right).

### Imaging results

#### Main effect of allocentric congruency

We first identified the brain regions activated by the main effect of allocentric congruency, i.e. “AlloIC (EgoC + EgoIC) > AlloC (EgoC + EgoIC)”. The right PosCG, extending anteriorly to the right PreCG, was significantly activated by the main effect contrast “AlloIC > AlloC” (collapsed over the factor of egocentric congruency; Fig. 2A and Table 1A). Based on the observed pattern of neural activity in the right PosCG (MNI: 48, −28, 50; Fig. 2A), the main effect of allocentric congruency in the right PosCG seems to be driven by the allocentric congruency effect only in the EgoC condition, but not in the EgoIC condition, i.e. an interaction rather than main effect. A conjunction analysis between the main effect of the allocentric congruency “AlloIC (EgoC + EgoIC) > AlloC (EgoC + EgoIC)” and the interaction effect “EgoC (AlloIC > AlloC) > EgoIC (AlloIC > AlloC)” further confirmed overlapping activations in the right PosCG (MNI: 48, −32, 50; Z = 4.69, 532 voxels), indicating that the significant main effect of allocentric congruency in the right PosCG is indeed driven by the interaction effect.

The reverse contrast, i.e., AlloC > AlloIC (collapsed over the factor of egocentric congruency), revealed no significant activation.

#### Main effect of egocentric congruency

No significant activation was revealed by the main effects of the egocentric congruency EgoIC vs. EgoC (collapsed over the factor of allocentric congruency).

#### Neural interaction between the egocentric and the allocentric congruency

The neural interaction contrast “EgoC (AlloIC > AlloC) > EgoIC (AlloIC > AlloC)” revealed significant activations in the right frontoparietal network, with maximum activation in the posterior portion of the right SPG (MNI: 24, −70, 48, BA7), extending dorsally to the right SMA and right superior frontal gyrus (SFG), and ventrally to the rolandic operculum (ROL) (Fig. 2B and Table 1B). No significant activations were observed in the reverse interaction contrast. Neural activity in the two single-source incongruent conditions (i.e., EgoC_AlloIC and EgoIC_AlloC) was both higher than in the congruent condition (i.e., EgoC_AlloC) and the double-source incongruent condition (i.e., EgoIC_AlloIC).

Since the allocentric and egocentric positions mismatched (i.e., one left and one right) in the EgoC_AlloIC and EgoIC_AlloC conditions, while matching (i.e., both left or both right) in the EgoC_AlloC and EgoIC_AlloIC conditions, the right SPG was involved in monitoring the mismatch in spatial locations relative to the egocentric and allocentric reference frame. To further investigate how the SPG communicates with the other brain regions as a “match vs. mismatch” detector, we further performed a PPI analysis with the interaction contrast “Mismatch (EgoC_AlloIC + EgoIC_AlloC) vs. Match (EgoC_AlloC + EgoIC_AlloIC)” as the psychological factor and with neural activity in the right SPG as the physiological factor. The right SPG showed significantly higher functional connectivity with the left middle temporal gyrus (MT) and the left ventral lateral inferior frontal gyrus (IFG) in the “Match” conditions (i.e., EgoC_AlloC and EgoIC_AlloIC) than in the “Mismatch” conditions (i.e., EgoC_AlloIC and EgoIC_AlloC) (Fig. 2C; Table 1C).

No significant modulation of neural coupling was found in the reverse direction (i.e., “Mismatch > Match”). The PPI analysis calculated functional connections between different brain areas, regardless of the height of neural activity. Therefore, even though the right SPG showed higher neural activity in the “Mismatch” than “Match” condition, the neural coupling between the right SPG and the ventral visual stream and the ventral lateral IFG was higher in the “Match” than “Mismatch” conditions.

#### Common neural correlates underlying the egocentric and allocentric Simon effects

**Figure 4:**
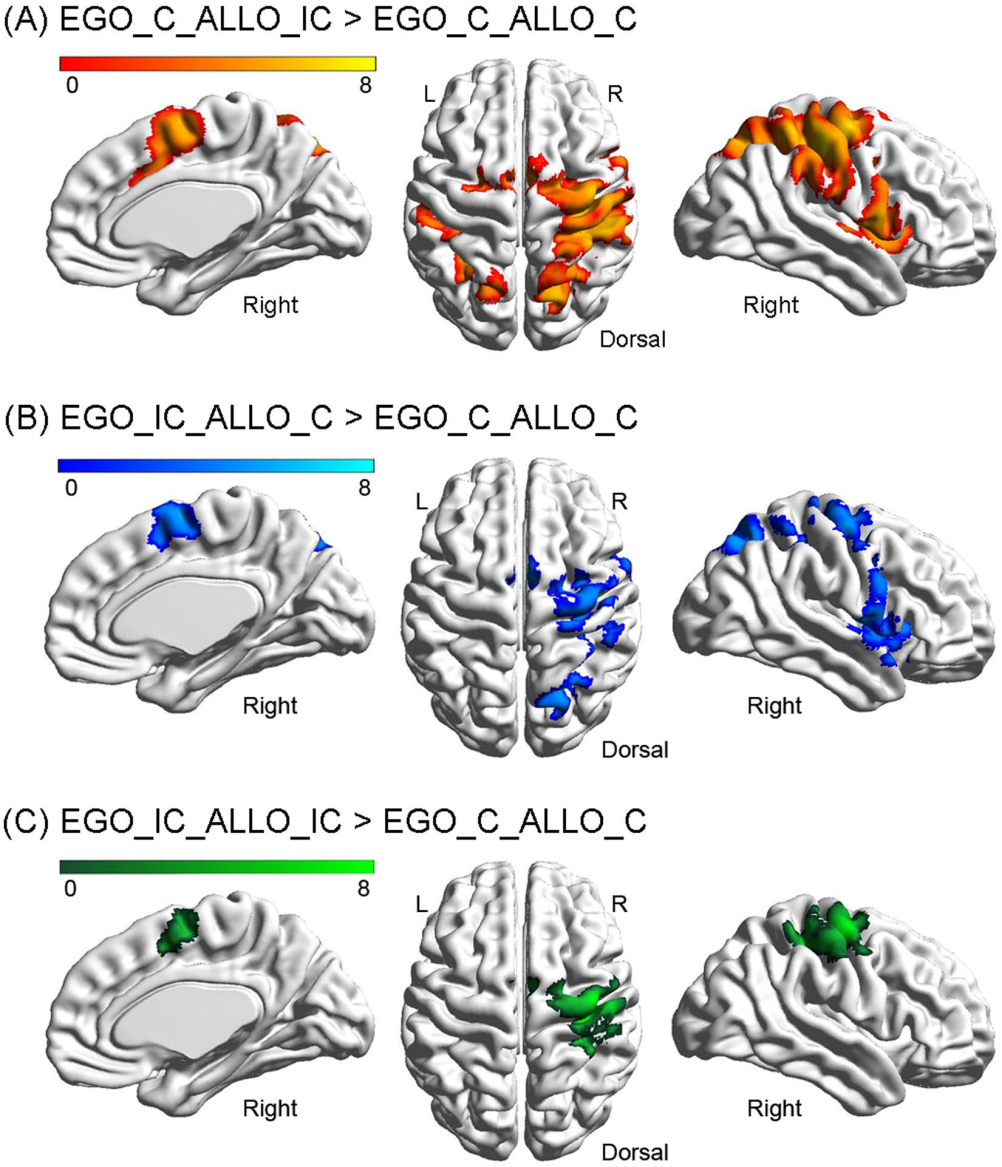
Neural activations in the three incongruent conditions as compared with the congruent condition. (**A**) Single-source allocentric incongruent condition, i.e., EgoC_AlloIC > EgoC_AlloC. (**B**) Single-source egocentric incongruent condition, i.e., EgoIC_ AlloC > EgoC_ AlloC. (**C**) Double-source incongruent condition, i.e., EgoIC_AlloIC > EgoC_AlloC.

Compared to the congruent condition (EgoC_AlloC), the single-source allocentric Simon conflict condition (EgoC_AlloIC) activated a dorsolateral frontoparietal network, including the bilateral posterior SPG, the bilateral IPG, the right PreCG, and the right PosCG (Fig. 4A and Table 3A). The single-source egocentric Simon conflict condition (EgoIC_AlloC) activated a similar frontoparietal network including the right SPG, the right angular gyrus (ANG), the right PreCG, and the right SMA (Fig. 4B and Table 3B). The double-source incongruent condition (EgoIC_AlloIC) activated a relatively smaller brain region, including the right PreCG and the right PosCG (Fig. 4C and Table 3C).

**Table 3:** Neural regions activated in the three incongruent conditions compared to the congruent condition.The coordinates (x, y, z) correspond to MNI coordinates. Displayed are the coordinates of the maximally activated voxel within a significant cluster as well as the coordinates of relevant local maxima within the cluster (in Italics).

Anatomical region	Side	Cluster peak (mm)	*t*-Score	*k*_E_ (voxels)
***(A) EgoC_AlloIC*** > ***EgoC_AlloC***				
Postcentral gyrus	R	46, −32, 52	7.88	7530
*Postcentral gyrus*	*R*	*48*, *−24*, *48*	*7*.*72*	
*Precentral gyrus*	*R*	*42*, *−10*, *58*	*7*.*50*	
Rolandic operculum	R	60, 6, 12	6.08	1702
*Insula lobe*	*R*	40, 0, 12	5.89	
Inferior parietal gyrus	L	−30, −48, 42	5.67	1071
*Superior parietal gyrus*	*L*	−18, −62, 56	4.88	
Cerebellum	L	−4, −68, −24	6.21	725
***(B) EgoIC_AlloC*** > ***EgoC_AlloC***				
Supplementary motor area	R	10, 0, 60	5.66	1926
*Precentral gyrus*	*R*	*42*, *−10*, *56*	*5*.*19*	
Insula lobe	R	44, 8, 0	4.54	964
*Precentral gyrus*	*R*	*58*, *6*, *20*	*4*.*37*	
Superior parietal gyrus	R	22, −68, 50	4.68	819
*Angular gyrus*	*R*	*32*, *−58*, *52*	*4*.*38*	
*Superior parietal gyrus*	*R*	*38*, *−48*, *58*	*3*.*85*	
Cerebellum	L	−4, −66, −22	6.6	774
***(C) EgoIC_AlloIC*** > ***EgoC_AlloC***				
Precentral gyrus	R	34, −24, 50	6.71	2288
*Postcentral gyrus*	*R*	*32*, *−34*, *48*	*4*.*99*	

To isolate the cortical regions that generally respond to the allocentric and egocentric Simon conflict whenever there is a conflict component irrespective of the spatial reference frame involved, a conjunction analysis was performed between the three incongruent conditions, i.e., the allocentric Simon conflict “EgoC (AlloIC > AlloC)” (Fig. 4A), the egocentric Simon conflict “AlloC (EgoIC > EgoC)” (Fig. 4B), and the Simon conflict based on both frames “EgoIC_AlloIC > EgoC_AlloC” (Fig. 4C). One cluster, extending from right PreCG to right SMA with maximum activation in the right PreCG, was significantly activated (Fig. 3A and Table 2A). Neural activity was significantly higher in all three incongruent conditions as compared to the congruent condition. Moreover, neural activity between the three incongruent conditions was comparable. The above results suggested that the right PreCG generally responded to the Simon effect irrespective of the spatial reference frame involved. Furthermore, since the height of neural activity in the double-source incongruent condition (i.e., EgoIC_AlloIC) was comparable to that in the two single-source incongruent conditions (i.e., EgoC_AlloIC and EgoIC_AlloC), the right PreCG did not respond to the egocentric and allocentric Simon conflict in an additive way.

#### Specific neural correlates underlying the allocentric Simon effect

To further isolate the specific neural correlates underlying the allocentric Simon conflict, we directly compared the three incongruent conditions, and localized the cortical regions that showed significantly higher neural activity in the EgoC_AlloIC condition compared to the other two incongruent conditions, by using the neural contrast “(EgoC_AlloIC) * 2 > [(EgoIC_AlloC) + (EgoIC_AlloIC)]”. One cluster, extending from the right PosCG to the right IPG, was significantly activated (Fig. 3B and Table 2B). Neural activity was higher in the allocentric Simon conflict condition (EgoC_AlloIC) than in the other two incongruent conditions (EgoIC_AlloC and EgoIC_AlloIC).

In addition, the two neural contrasts “(EgoIC_AlloC) * 2 > [(EgoC_AlloIC) + (EgoIC_AlloIC)]” and “(EgoIC_AlloIC) * 2 > [(EgoC_AlloIC) + (EgoIC_AlloC)]” were used to localize the specific neural correlates underlying the single-source egocentric Simon conflict and the double-source conflict induced by both frames, respectively. However, no significant activation was found.

## Discussion

By orthogonally manipulating the Simon effect based on allocentric and egocentric spatial reference frames, in this fMRI study, we revealed novel neural correlates underlying the allocentric Simon effect and its neural interactions with the egocentric Simon effect. Behaviorally, both the classic egocentric Simon effect and the allocentric Simon effect were observed (Fig. 1C). Neurally, we found three different patterns of neural interactions between the allocentric and egocentric Simon effect (Figs 2 and 3). In the following paragraphs, we are going to focus our discussion on the three different patterns of neural interaction and the underlying theoretical implications.

### General neural mechanisms underlying the Simon effect

The right PreCG, extending to the right SMA, showed significantly increased neural responses in all the three incongruent conditions, compared to the congruent condition (Fig. 3A), suggesting that these areas were generally activated by Simon conflicts, irrespective of the type of spatial reference frame involved.

It has been reported that the PreCG was generally associated with various types of spatial compatibility tasks^31–36^. In particular, when participants were asked to respond to stimuli presented in either the right or left visual field with either the ipsilateral (compatible conditions) or the contralateral hand (incompatible conditions), the activation of PreCG significantly increased in the incompatible conditions as compared to the compatible conditions^32,34^. Furthermore, previous evidence has shown that single-pulse Transcranial Magnetic Stimulation (TMS) over the PreCG induced facilitatory responses in the incongruent condition, suggesting a causal-functional role of the PreCG in conflict resolution^34^. Similar to the PreCG, previous evidence from a large body of neuroimaging research on a variety of different conflict tasks, such as the Simon, Stroop, and Eriksen flanker tasks, points to a general role of the SMA in conflict resolution^15,17,19,35–39^. For example, it has been suggested that the SMA is commonly involved in both the Stroop and Simon conflicts^15^. Also, by asking subjects to identify shapes based on form-from-motion perception within a randomly moving dot field while ignoring the motion direction or stimulus location, Wittfoth (2006) found that the two variations of the Simon task shared neural activations in pre-SMA/SMA during conflict resolution^19^.

In the Simon-type tasks, the task-irrelevant position automatically activates the ipsilateral response through a direct route, whereas the task-relevant stimulus feature activates the correct response via a controlled route^40^. To successfully perform the current task, especially in the incongruent conditions, participants were required to override contextual influences on the motor system emerging from irrelevant target representations, within the egocentric or the allocentric reference frame or both, in order to maintain unbiased spatial representations of the response hand. The PreCG, comprised of premotor and primary motor area, is considered as a crucial neural area for planning, selecting, and executing responses^41–45^. Evidence from previous Simon type tasks suggests that the Simon conflict is associated with neural responses in the PreCG that are involved in inhibiting task-irrelevant representations^46–52^. On the other hand, the SMA is associated with executive control of motor outputs, in particular with selecting the appropriate response between different response alternatives^53–55^. In the current study, neural activations in the PreCG and SMA might be involved in voluntarily selecting the task-relevant correct responses while inhibiting the motor tendency induced by the task-irrelevant spatial positions. Taken together, the present results not only confirm but also extend previous evidence by showing that the right PreCG and the right SMA subserved the maintenance of unbiased response representations by resolving the conflicts from contextual information, irrespective of whether the contextual information is based on the allocentric or the egocentric reference frame.

Moreover, our results showed that the right PreCG and SMA do not respond to the Simon conflict in an additive way: the height of neural activity in the double-source incongruent (EgoIC_ AlloIC) condition was comparable to that in the two single-source incongruent (EgoC_ AlloIC and EgoIC_ AlloC) conditions (Fig. 3A). Therefore, this pattern of results supports the model that the allocentric and egocentric Simon conflicts occur based on a common abstract spatial coordinate system in which the allocentric and egocentric positions are coded by the same abstract coordinate (Fig. 1B, right). It is widely accepted that spatial locations of stimuli are automatically encoded, even though they may be entirely irrelevant for an ongoing task^56,57^. In particular, the spatial location of an object could be automatically encoded upon its appearance for both the egocentric and the allocentric reference^10,58–60^. From an evolutionary point of view, an economical solution to simultaneously code the allocentric and egocentric locations of an object in the human brain is to use one common abstract coordinate, which allows different reference frames to converge onto a unified spatial coordinate system. Therefore, during sensorimotor transformation, spatial representations of discrete sensory stimuli are transformed into a common high-level abstract coordinate system^5,61,62^. In the double-source incongruent (EgoIC_ AlloIC) condition of the present study, the egocentric and allocentric locations of the target share the same coordinate in the high-level abstract coordinate system (Fig. 1B right) even though that one is coded as left while the other is coded as right (Fig. 1A). The PreCG and SMA may be generally involved in inhibiting the incongruent spatial codes in the abstract coordinate system, rather than the discrete allocentric and egocentric representations per se. Since the single-source and the double-source incongruent conditions refer to the same abstract incongruent coordinates, it does not make any difference for the PreCG and SMA to inhibit the same abstract incongruent code between the two conditions.

### Specific neural mechanisms underlying the allocentric Simon effect

The caudal part of the PosCG (BA2) was specifically involved in the allocentric Simon effect, by showing increased neural activity in the allocentric Simon conflict (EgoC_ AlloIC), compared to the egocentric Simon conflict (EgoIC_ AlloC), and the Simon conflict induced by both frames (EgoIC_ AlloIC) (Figs 2A and 3B). In the allocentric reference frame, object positions are primarily represented relative to the configurational properties of objects, such as the relationships among different components of one object or different objects in the environment. It has been demonstrated that allocentric spatial representations subserve the conscious perception/identification of objects and are represented mainly along the ventral visual stream^3,63–68^. In order to make goal-directed actions towards external objects, however, the discrete object representations in the ventral visual stream need to be transformed to the corresponding sensorimotor representations in the frontoparietal network via the dorsal visual stream^69–71^. Previous evidence from our lab showed that allocentric judgments on object locations activated both the ventral visual areas and the dorsal frontoparietal network^72^. Moreover, part of the right PosCG was involved in resolving spatial conflicts caused by task-irrelevant allocentric representations during egocentric judgment tasks^60^. In the current study, we further revealed that the right PosCG was specifically involved in the Simon type conflicts between the task-irrelevant allocentric representations and the response hand (Fig. 3B).

The PosCG (primary somatosensory cortex) is reported to be the confluence of several interconnected sensorimotor processes. These processes depend on visual, tactile, and motorial information to generate and execute goal-directed actions, such as visually guided reaching and grasping movements^64,73–76^. Moreover, previous neuroimaging studies on various types of the Simon effect suggest that the right postcentral area contains subregions which contribute to conflict monitoring^15,16,19,77,78^. For instance, in a motion-based Simon task, in which the task-irrelevant motion direction of the target was either consistent or inconsistent with the response hand, the right PosCG was significantly activated^19^. Since the task-irrelevant motion direction in that study was defined by the relative positions between the moving dots, i.e., allocentric information, enhanced neural activity in the right PosCG in the incongruent condition (compared to the congruent condition) indicates the functional role of the right PosCG in resolving conflict specifically caused by irrelevant allocentric representations.

### Specific mechanisms underlying the single-source Simon effect

The neural interaction contrast “EgoC (AlloIC > AlloC) > EgoIC (AlloIC > AlloC)” revealed significant activations in a dorsolateral frontoparietal network, including the right posterior SPG (BA7) extending into the right SMA, the right SFG, and the right IFG (Fig. 2B and Table 1B). This right lateralized dorsolateral frontoparietal network was specifically involved in the single-source Simon effect, as indicated by increased neural activity when one type of spatial representation was incongruent while the other type was congruent with the response hand (i.e., in the EgoC_ AlloIC and EgoIC_ AlloC conditions), compared to when both spatial representations were congruent (EgoC_ AlloC) or both incongruent (EgoIC_ AlloIC) with the response hand (Fig. 2B).

Neural activations in the frontoparietal network have been shown to increase significantly during conflict processing^79–81^. For example, significant effects of interference were found in the frontoparietal network, and their activation probably reflects response conflicts, cognitive control, and conflict detection^82–86^. Among the frontoparietal network, the right posterior SPG is critical for spatial location representations^63,87–91^ and the integration of spatial information from different reference frames during the localization of objects^92–96^. The right SPG also plays an essential role in manipulating spatial stimulus-response compatibility^33,97–99^. Besides, the right frontal brain regions have been suggested to be involved in the maintenance of spatial information during the processing of spatial relations^100^. Therefore, consistent with previous evidence, increased neural activity in the right frontoparietal network during the single-source Simon effect of the present study might be related to monitoring spatial information from multiple coordinate systems. Specifically speaking, in the current study, the allocentric and egocentric location of the target could either match (i.e., on the same side in the EgoC_ AlloC and the EgoIC_ AlloIC condition) or mismatch (i.e., on different sides in the EgoC_ AlloIC and EgoIC_ AlloC condition) (Fig. 1A). Based on the observed pattern of neural activity in the right SPG, which is the maximum activation in the frontoparietal network, we found significantly higher neural activity in the two “Mismatch” conditions (EgoC_ AlloIC and EgoIC_AlloC) than the two “Match” conditions (EgoC_ AlloC and EgoIC_AlloIC) (Fig. 2B). According to our hypothesis, upon integrating spatial information from egocentric and allocentric reference coordinate systems into the common abstract spatial coordinate system, the right dorsolateral frontoparietal network might function as a mismatch detector between various sources of spatial information. This mismatch detector will be activated and generate mismatch signals whenever the allocentric and egocentric positions of the same object are on opposite sides.

Moreover, based on the results of the PPI analysis, the right SPG (the maximum neural activation in the frontoparietal network) showed enhanced functional interactions with the left ventral MT and the left ventral lateral IFG in the “Match” conditions, compared to the “Mismatch” conditions (Fig. 2C). Visual processing in the human brain is anatomically organized along two distinct streams: a dorsal stream, which is originating from the striate cortex and projecting to the posterior parietal gyrus, and a ventral stream, which is originating from the striate cortex and reaching the inferotemporal cortex^67,101–103^. Besides, these two visual streams are not only anatomically but also functionally distinct. External visual inputs are transformed into perceptual representations in the ventral visual stream, which enables the recognition of objects as well as their spatial relations, while visual information was transformed into the sensorimotor representations in the dorsal visual stream, which supports the planning and online control of visually guided actions in the environment^1,67,102,104^. Further anatomical studies showed that visual projections in the dorsal stream are extended from posterior parietal gyrus finally into the principal sulcus (BA 46) of the dorsolateral prefrontal cortex^105–108^, whereas projections in the ventral stream is extended from inferotemporal gyrus ultimately into the inferior convexity (BA 12 and 45) of the ventrolateral prefrontal cortex^109–111^. Functionally, face processing activates the right IFC in the ventral stream, whereas location processing activates the superior frontal region in the ventral stream^112^. Indeed, the ventral stream projections to the MT and the IFC are exquisitely prepared to serve as an interface between vision and cognition^113^. Therefore, in the current study, the enhanced neural coupling between the right SPG in the dorsal and the ventral visual stream in the “Match” conditions may contribute to representing a common abstract spatial representation where egocentric and allocentric reference frame information converges, i.e., activating a general concept of left and right.

Please also note, the current neural activations in the dorsolateral frontoparietal network are mostly right lateralized (Fig. 2B and Table 1B). Numerous studies on healthy subjects and neurosurgical, brain-injured patients consistently reported that the right hemisphere was specialized in the visuospatial analysis of the external world^114–117^. For example, unilateral inattention and spatial neglect occur much more frequently after right than left hemisphere (parietal, frontal, thalamic, basal ganglia) damage, in particular for lesions located in the right temporal-parietal and occipital junction^118–120^. Moreover, the right hemisphere has been found to be superior in discriminating the directional orientation of the body as well as body-part positional relationships^115,117,121,122^. In most of the previously reported studies on visuospatial processing, the spatial representations involved were based on one specific spatial coordinate system. In the current study, we further revealed that the right dorsolateral frontoparietal network monitors spatial representations not only in a specific spatial coordinate system but also in the high-level abstract coordinate map.

## Conclusion

To summarize, by orthogonally combining the Simon effect based on allocentric and egocentric reference frames, we revealed general and specific neural correlates underlying egocentric and allocentric Simon conflicts. We found that the right PreCG, extending to the right SMA, is generally involved in the Simon conflict between spatial information and response code, irrespective of the spatial reference frame involved. Furthermore, right PosCG is specifically involved in the Simon conflict induced by task-irrelevant allocentric representations. Finally, our results suggest that the right dorsolateral frontoparietal network, including the right posterior SPG extending into the right IFG, may function as a mismatch detector to monitor the congruency between the two sources of spatial information with regard to the allocentric and egocentric reference frames.
